# Multidrug Resistance and *mup*A-Mediated Mupirocin Resistance in Clinical Coagulase-Negative Staphylococci

**DOI:** 10.3390/pathogens15070745

**Published:** 2026-07-15

**Authors:** Catarina Freitas, José Eduardo Pereira, Eliana Costa, Olga Alves, Gilberto Igrejas, Patrícia Poeta, Vanessa Silva

**Affiliations:** 1 Microbiology and Antibiotic Resistance Team (MicroART), Department of Veterinary Sciences, University of Trás-os-Montes and Alto Douro (UTAD), 5000-801 Vila Real, Portugal; catarinairfreitas@gmail.com (C.F.);; 2 Associated Laboratory for Green Chemistry (LAQV-REQUIMTE), University NOVA of Lisbon, 2829-516 Caparica, Portugal; 3 Associated Laboratory for Animal and Veterinary Science (AL4AnimalS), University of Trás-os-Montes and Alto Douro (UTAD), 5000-801 Vila Real, Portugal; 4 CECAV—Veterinary and Animal Research Centre, University of Trás-os-Montes and Alto Douro (UTAD), 5000-801 Vila Real, Portugal; 5 Clinical Pathology Department, Hospital Centre of Trás-os-Montes and Alto Douro, 5000-508 Vila Real, Portugal; 6 Department of Genetics and Biotechnology, University of Trás-os-Montes and Alto Douro (UTAD), 5000-801 Vila Real, Portugal; 7 Functional Genomics and Proteomics Unit, University of Trás-os-Montes and Alto Douro (UTAD), 5000-801 Vila Real, Portugal

**Keywords:** coagulase-negative staphylococci, antimicrobial resistance, biofilm formation, multidrug resistance, *mec*A, nosocomial infections, mupirocin, *mup*A

## Abstract

Coagulase-negative staphylococci (CoNSs) are major opportunistic pathogens in healthcare settings, particularly affecting immunocompromised patients and those with indwelling medical devices. Their growing antimicrobial resistance and ability to form biofilms present significant therapeutic challenges. This study analyzed 148 clinical CoNS isolates to determine species distribution, antimicrobial resistance, resistance genes, and biofilm production. *Staphylococcus epidermidis* was the most prevalent species (49.3%), followed by *Staphylococcus hominis* (27.0%) and *Staphylococcus capitis* (6.8%). Penicillin (83.8%), erythromycin (72.3%), and cefoxitin (66.2%) showed the highest resistance rates. Notably, 60.8% of isolates were resistant to fusidic acid and 45.3% to clindamycin, with inducible resistance in 12.1%. Among aminoglycosides, tobramycin resistance (45.3%) was most frequent. Resistance to ciprofloxacin and trimethoprim–sulfamethoxazole reached 45.9%, while mupirocin resistance was 17.6%. Among isolates resistant to penicillin and/or cefoxitin (*n* = 128), the *mec*A gene was detected in 69.5%; the mecC gene was absent. Only 38.5%% of mupirocin-resistant isolates carried the *mup*A gene. Trimethoprim resistance was mainly associated with *dfr*A (60.3%) and *dfr*G (14.7%). Biofilm super-producers accounted for 64.9% of isolates and non-producers for 7.4%, with no significant link between biofilm formation and antibiotic resistance. These findings reinforce the clinical relevance of multidrug-resistant, mupirocin-resistant, and biofilm-forming CoNSs, underscoring the need for improved surveillance and infection control.

## 1. Introduction

Coagulase-negative staphylococci (CoNSs) are a diverse group of Gram-positive bacteria that constitute part of the normal microbiota of humans and animals [1]. Historically considered non-pathogenic, they have increasingly been recognized as opportunistic pathogens, particularly in hospital environments [2,3]. These bacteria are classified based on their inability to produce coagulase, distinguishing them from Coagulase-positive staphylococci (CoPSs), which are generally more virulent [4]. Despite their lower virulence, CoNSs have become significant agents of nosocomial infections, largely due to the widespread use of medical devices and immunosuppressive treatments [5].

Among CoNSs, *Staphylococcus epidermidis* is the species most frequently associated with healthcare-associated infections, particularly those involving indwelling medical devices [6]. Other clinically relevant species, such as *S. haemolyticus*, *S. hominis*, and *S. saprophyticus*, have been implicated in conditions ranging from bacteremia to urinary tract infections [5]. A key factor contributing to the persistence of CoNS infections is their ability to form biofilms, structured microbial communities that facilitate adhesion to surfaces and confer protection against the host immune system and antimicrobial agents. Biofilm formation on medical implants, such as catheters and prosthetic devices, significantly complicates treatment and eradication, increasing the risk of chronic infections and therapeutic failure [6].

CoNSs also exhibit increasing antimicrobial resistance, which further complicates their clinical management. Many isolates have developed resistance to multiple antibiotic classes, including β-lactams, aminoglycosides, and fluoroquinolones [7]. Of particular concern is the presence of the *mec*A gene, which confers resistance to methicillin and has led to the widespread emergence of methicillin-resistant CoNSs (MR-CoNSs) in healthcare settings [8]. These multidrug-resistant strains not only complicate treatment options but also serve as reservoirs of resistance genes, with the potential for horizontal gene transfer to more virulent species, such as *Staphylococcus aureus*. Studies have shown that CoNS strains isolated from hospital environments exhibit high levels of resistance to multiple antibiotics, underscoring the urgent need for judicious antimicrobial use and stringent infection control measures to prevent nosocomial dissemination of these pathogens [1].

Given these concerns, this study aims to characterize the antimicrobial resistance profiles and biofilm-forming capacity of CoNS isolates from patients at the Hospital Center of Trás-os-Montes and Alto Douro.

## 2. Results

### 2.1. Distribution of Species by Gender and Age Group

**Figure 1:**
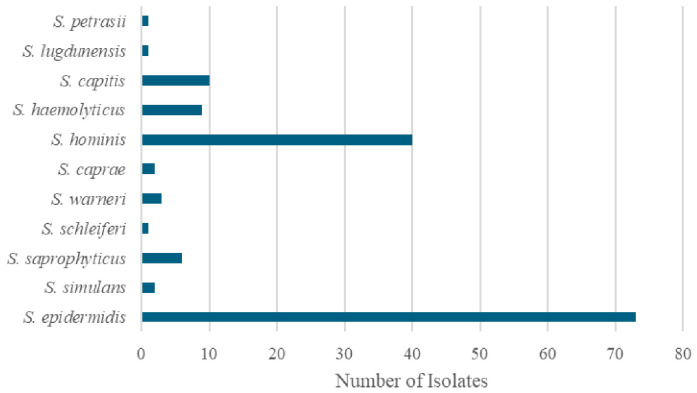
Number of isolates of each CoNS species.

A total of 148 CoNS isolates were analyzed and distributed among different species. The most frequently identified species was *S. epidermidis* (49.3%, *n* = 73), followed by *S. hominis* (27.0%, *n* = 40) and *S. capitis* (6.8%, *n* = 10) (Figure 1). The remaining species, including *Staphylococcus simulans*, *S. saprophyticus*, *Staphylococcus schleiferi*, *Staphylococcus warneri*, *Staphylococcus caprae*, *Staphylococcus haemolyticus*, *Staphylococcus lugdunensis*, and *Staphylococcus petrasii*, exhibited a distribution frequency ranging from one to nine isolates (0.68–6.1%) (Figure 1).

**Figure 2:**
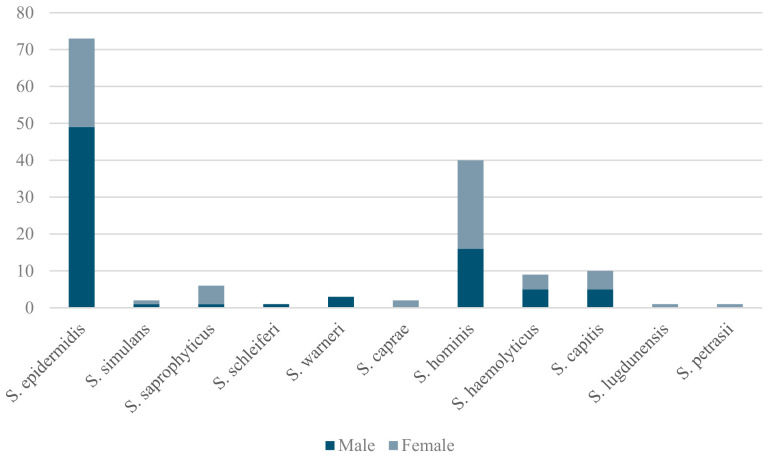
Distribution of the different CoNS species by gender.

Regarding gender distribution, 81 isolates (54.7%) were obtained from male patients, while 67 (45.3%) were from female patients. *S. epidermidis* was more prevalent in male patients, representing 67.1% (*n* = 49) of the isolates, while 32.9% (*n* = 24) belonged to female patients (Figure 2). However, *S. hominis* was more frequent in female patients (60%) than in male patients (40%) (Figure 2). Conversely, *S. saprophyticus* and *S. caprae* were predominantly detected in female patients. A statistically significant but weak association was found between patient gender and CoNS species distribution (χ^2^ = 10.038, *p* = 0.040, Cramér’s V = 0.260). To ensure the validity of the chi-square test, species for which expected cell frequencies fell below five in at least one gender group were grouped into a single “Other CoNSs” category, comprising *S. saprophyticus*, *S. warneri*, *S. simulans*, *S. caprae*, *S. schleiferi*, *S. lugdunensis*, and *S. petrasii* (*n* =16).

**Figure 3:**
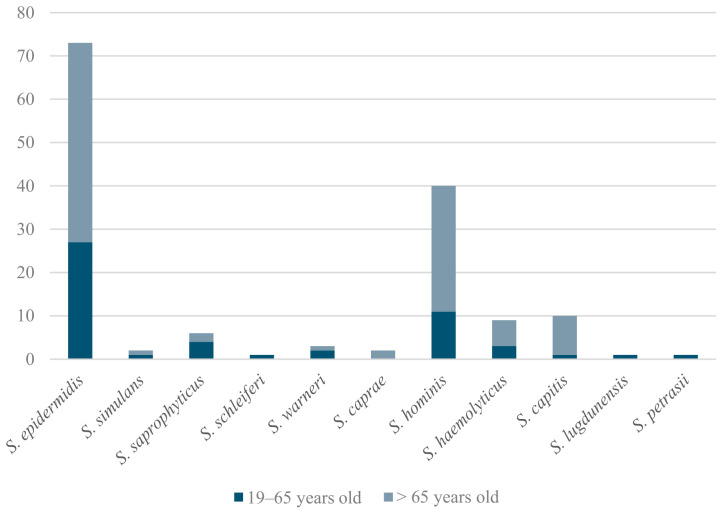
Distribution of the different CoNS species by age.

With respect to age distribution, most isolates (64.9%) were obtained from patients over 65 years old, while 35.1% were from patients aged 19 to 65 (Figure 3). No isolates were identified in patients under 18 years old (Figure 3). In both age groups, *S. epidermidis* was the predominant species, detected in 46 patients over 65 and in 27 patients aged 19 to 65. Other species, such as *S. hominis*, were also more common in younger adults. An analysis of the potential correlation between patient age and CoNS species revealed no statistically significant associations (χ^2^ = 6.501, *ρ* = 0.165), suggesting that age does not have a substantial impact on species distribution.

### 2.2. Phenotypic and Genotypic Characterization of Antimicrobial Resistance

The antimicrobial resistance profiles of the 148 CoNS isolates were evaluated, incorporating both phenotypic resistance patterns and genotypic determinants. The analysis revealed a high prevalence of resistance across multiple antibiotic classes, with 81.1% of isolates classified as multidrug-resistant (MDR), exhibiting resistance to three or more antibiotic classes.

#### 2.2.1. Phenotypic Resistance Patterns

**Figure 4:**
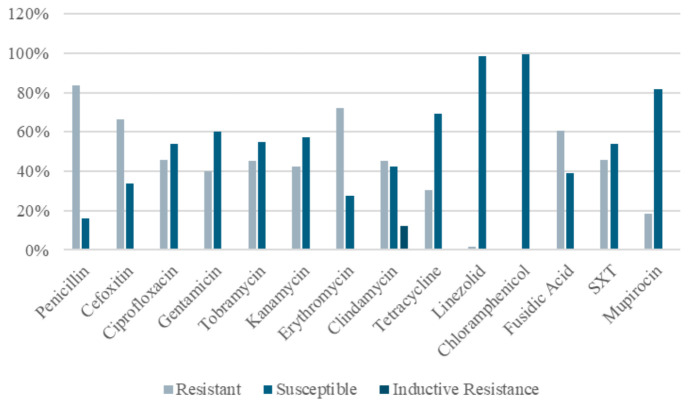
Resistance and susceptibility to antibiotics in CoNS isolates.

The highest resistance rates were observed for penicillin (83.8%), followed by erythromycin (72.3%) and cefoxitin (66.2%) (Figure 4). Resistance to fusidic acid was also notable (60.8%). Additionally, 45.3% of isolates were resistant to clindamycin, with 12.1% showing inducible resistance (Figure 4). Among aminoglycosides, resistance was observed in 45.3% of isolates for tobramycin, 42.6% for kanamycin, and 39.9% for gentamicin. Moderate resistance levels were found for ciprofloxacin (45.9%), trimethoprim–sulfamethoxazole (45.9%), and tetracycline (30.4%). In contrast, linezolid resistance was low (1.4%), and chloramphenicol resistance was rare, with only one resistant isolate (Figure 4). Mupirocin resistance was observed in 26 isolates (17.6%), all of which showed no zone of inhibition, consistent with high-level resistance according to CLSI criteria. An additional seven isolates presented inhibition zones between 1 and 29 mm, below the EUCAST breakpoint of 30 mm established for *S. aureus* but were not classified as resistant given the absence of validated interpretive criteria for CoNSs (Figure 4).

Significant associations (*p* < 0.05) were identified between species and resistance to multiple antibiotics, including penicillin, cefoxitin, ciprofloxacin, gentamicin, and trimethoprim–sulfamethoxazole (Table 1). *S. haemolyticus* exhibited the highest resistance rates across all five antibiotics, reaching 100% for penicillin, ciprofloxacin, gentamicin, and trimethoprim–sulfamethoxazole, and 88.9% for cefoxitin. *S. epidermidis* also showed high resistance rates, particularly for penicillin (91.8%) and cefoxitin (79.5%), with moderate resistance to ciprofloxacin (56.2%), gentamicin (52.1%), and trimethoprim–sulfamethoxazole (57.5%). In contrast, *S. hominis* presented lower resistance rates for cefoxitin (50%), ciprofloxacin (35%), gentamicin (27.5%), and trimethoprim–sulfamethoxazole (37.5%), despite high penicillin resistance (85%). *S capitis* showed a distinct profile, with high penicillin resistance (80%) but markedly lower rates for cefoxitin (40%), ciprofloxacin (20%), and trimethoprim–sulfamethoxazole (10%), and no resistance detected for gentamicin. No significant associations were found for tetracycline (*p* = 0.312), fusidic acid (*p* = 0.285), mupirocin (*p* = 0.123), and chloramphenicol (*p* = 1.000), indicating a more homogeneous distribution of resistance across species (Table 1).

#### 2.2.2. Genotypic Resistance Determinants

Genotypic analysis was conducted to identify resistance genes corresponding to the observed phenotypic patterns. Isolates resistant to penicillin and/or cefoxitin (*n* = 128) were screened for the mecA gene, which was detected in 89 (69.5%). Considering isolates resistant specifically to cefoxitin, mecA was absent in 12 of 58 (20.7%) *S. epidermidis* and in none of the 25 (0%) *S. hominis* isolates. The *mec*C gene, examined in mecA-negative isolates, was absent in all cases. The *bla*Z gene was present in only 21.8% of penicillin-resistant isolates (Table S1).

Regarding macrolide and lincosamide resistance, 27.3% of isolates carried the *msr*A/B gene, while 5.5% harbored *mph*C. Among tetracycline resistance isolates, none carried the *tet*O gene. For aminoglycoside resistance, 71.4% of isolates carried the *aac*(6′)-Ie-*aph*(2″)-Ia gene. The chloramphenicol resistance genes *cat*_PC194_, *cat*_PC221_, *cat*_PC223_, *fex*A, and *fex*B were not detected (Table S1).

The *dfr*A, *dfr*D, *dfr*G, and *dfr*K genes were analyzed for trimethoprim–sulfamethoxazole resistance. The *dfr*A gene was present in 60.3% of isolates, while *dfr*G was detected in 14.7%; *dfr*D and *dfr*K were absent. Among mupirocin-resistant isolates, the *mup*A gene was detected in 10 of 26 (38.5%), all of which showed no zone of inhibition. The remaining 16 *mup*A-negative isolates were predominantly *S. epidermidis* (*n* = 11), followed by *S. hominis* (*n* = 3), *S. haemolyticus* (*n* = 1), and *S. capitis* (*n* = 1) (Table S1).

Chi-square analysis revealed that certain species carried specific resistance genes more frequently, particularly *msr*A/B, *dfr*A, *dfr*G, and *mup*A. However, the distribution of *mec*A, *bla*Z, *mph*C, and *acc*(6′)-Ie-*aph*(2″)-Ia was more random among species.

### 2.3. Biofilm Formation

**Figure 5:**
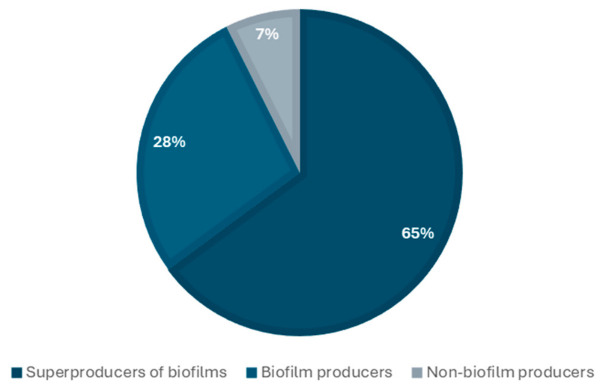
Percentage distribution of coagulase-negative Staphylococci (CoNS) isolates according to their ability to form biofilm. The isolates were classified as biofilm super-producers, biofilm producers, and non-biofilm producers based on the quantification of biofilm biomass.

Biofilm formation capacity was assessed using a standard reference strain of S. epidermidis (ATCC^®^ 35984) as a positive control on every plate, with each isolate’s biofilm formation expressed as a percentage of the reference strain’s OD570 value on the same plate. Isolates were classified as non-producers (below 50% of the reference value), producers (50% to 100%), or super-producers (above 100%). Overall, 96 isolates (64.9%) were classified as super-producers, 42 (27.7%) as producers, and 11 (7.4%) as non-producers (Figure 5).

Analysis of variance (ANOVA) did not indicate significant differences in biofilm formation between species. Additionally, a Mann–Whitney U test found no significant association between biofilm formation and antibiotic resistance, suggesting that resistance to the antibiotics tested does not directly influence biofilm production. However, strong associations (*p* = < 0.05) were identified between certain resistance patterns, particularly erythromycin–clindamycin, clindamycin–trimethoprim–sulfamethoxazole, cefoxitin-penicillin, gentamicin–tobramycin, ciprofloxacin–cefoxitin, kanamycin–tobramycin, and more, indicating possible co-resistance or shared genetic mechanisms (Table S2).

## 3. Discussion

The distribution of CoNS species in this study is consistent with previous reports, with *S. epidermidis* being the most frequently isolated species, followed by *S. hominis* and *S. capitis* [9]. The predominance of *S. epidermidis* aligns with its well-documented role in nosocomial infections, particularly those associated with medical devices [10]. Interestingly, a statistically significant, though weak, association was found between patient gender and CoNS species distribution (Cramér’s V = 0.260), suggesting that *S. hominis* and *S. caprae* may exhibit a higher prevalence in female patients; the modest effect size warrants caution in interpretation. In contrast, patient age showed no significant association with species distribution.

The multidrug resistance rate of 81.1% observed among isolates is consistent with reports identifying CoNS as critical reservoirs of antimicrobial resistance in hospital settings, with rates above 70% previously described for *S. epidermidis* and *S. haemolyticus* specifically [7,11], reinforcing the role of CoNS as a vector for horizontal gene transfer of resistance genes under the selective pressure typical of health care settings.

The resistance rates observed for penicillin (83.8%) and cefoxitin (66.2%) are consistent with previous studies identifying β-lactam resistance as among the most prevalent characteristics in CoNS isolates [12]. Penicillin resistance likely reflects the widespread use of this antibiotic and the well-documented production of β-lactamase by staphylococci. However, only 21.8% of penicillin-resistant isolates carried the *bla*Z gene, suggesting a contribution from other mechanisms, such as alterations in penicillin-binding proteins. The presence of *mec*A in 46 of 58 (79.3%) cefoxitin-resistant *S. epidermidis* isolates and in all 25 (100%) cefoxitin-resistant *S. homini*s isolates supports this gene as a major determinant of methicillin resistance in CoNS, consistent with previously reported detection rates of 60% to 80% [13]. Considering all isolates screened for *mec*A in this study (resistant to penicillin and/or cefoxitin, *n* = 128), the gene was detected in 89 (69.5%). Twelve *S. epidermidis* isolates (20.7%) were phenotypically resistant but mecA-negative, a discrepancy not observed in *S. hominis*. Cefoxitin susceptibility for these isolates was determined by the Vitek 2 system rather than disc diffusion, precluding confirmation of which EUCAST breakpoint for *S. epidermidis* (R < 27 mm) or the generic threshold applied when CoNSs are not identified to species level (R < 25 mm) was used, nor to examine whether these isolates cluster near the breakpoint, as has been done for other CoNS species using zone-diameter data [14]. The absence of *mec*C, predominantly found in other staphylococcal species such as *S. scuri* and *S. xylosus*, is inconsistent with its rarity in clinical CoNS isolates [15,16].

Macrolide and lincosamide resistance was also very high, with resistance to erythromycin present in 72.3% of isolates and clindamycin resistance detected in 45.3%, including 12.1% exhibiting inducible resistance, consistent with earlier reports of erythromycin resistance in 60% of hospital-acquired CoNS isolates [17,18]. *msr*A/B (27.3%) points to a considerable efflux-mediated contribution to the macrolide phenotype [19], while the low prevalence of *mph*C (5.5%) suggests that other mechanisms, such as ribosomal target modification, may also be involved.

Aminoglycoside resistance was also notable, with rates of 45.3% for tobramycin, 42.6% for kanamycin, and 39.9% for gentamicin, similar to previous reports [17]. The detection of *aac*(6′)-Ie-*aph*(2″)-Ia in 71.4% of aminoglycoside-resistant isolates is consistent with its established role as the major aminoglycoside resistance determinant in staphylococci [20,21].

The ciprofloxacin resistance rate observed here (45.9%) falls within the range reported in the literature (14–63.7%) across different studies and geographical regions [22,23,24], highlighting the variability in resistance patterns to this antibiotic.

Trimethoprim–sulfamethoxazole resistance was detected in 45.9% of the isolates, with the *dfr*A gene identified in 60.3% of resistant strains and *dfr*G in 14.7%, consistent with previously reported rates (46.5% to 72%) and the established predominance of these genes in CoNS [25,26,27,28,29]. The absence of *dfr*D and *dfr*K further supports their infrequency among staphylococcal species [28].

Resistance rates were much lower in linezolid (1.4%) and for chloramphenicol (a single resistant isolate), consistent with the low global prevalence of linezolid non-susceptibility in CoNS relative to other Gram-positive pathogens [30]. The lack of *fex*A and *fex*B genes further supports the continued rarity of chloramphenicol resistance in clinical CoNS.

Mupirocin resistance remains a significant clinical issue in nosocomial environments where it is commonly utilized as part of decolonization strategies [31]. In hospital settings, mupirocin resistance has been reported as rates ranging from 10% to 30%, and higher rates have been reported in regions with heavy mupirocin usage for infection control [32,33,34,35], a range consistent with the present findings. It should be noted that EUCAST and CLSI interpretive criteria for mupirocin were established for *S. aureus*, and their application to CoNSs constitutes a methodological extrapolation, as species-specific breakpoints for this group have not been validated [36,37]. In this regard, seven isolates in the present study presented an inhibition zone between 1 and 29 mm, below the EUCAST breakpoint of 30 mm but were not classified as resistant given this limitation. The *mup*A gene was detected in 10 of the 26 phenotypically resistant isolates (38.5%), consistent with the established role of this gene in mediating high-level mupirocin resistance through acquisition of an alternate isoleucyl-tRNA synthetase with reduced affinity for mupirocin [38]. This prevalence is in agreement with previous reports: Ref. [39] identified the *mup*A gene in 30% of *S. epidermidis* clinical isolates, while Ref. [40] reported a slightly higher prevalence of 45%. Conversely, markedly lower prevalence rates, ranging from 0% to approximately 10%, have been documented in other settings [32,34,41,42], reflecting the considerable variability in the distribution of this resistance determinant across different clinical contexts and geographical regions, likely attributable to differences in mupirocin usage patterns and genetic diversity among CoNS isolates. The remaining 16 *mup*A-negative isolates with no zone of inhibition represent a genotype/phenotype discordance that warrants consideration of both methodological and biological explanations. From a methodological standpoint, the primers used for *mup*A detection were originally described and validated exclusively in *S. aureus* [43], and their sensitivity in CoNS species has not been formally assessed, raising the possibility of false-negative results attributable to sequence divergence between *mup*A variants circulating in CoNS and the primer target region. From a biological standpoint, the absence of *mup*A in phenotypically resistant isolates may reflect the presence of alternative resistance determinants not investigated in this study, most notably *mup*B, a distinct locus encoding an alternate isoleucyl-tRNA synthetase that has also been documented as a cause of high-level mupirocin resistance [38]. Although *mup*A-negative high-level mupirocin resistance in *S. epidermidis* has been documented, it is considered rare and was identified by real-time PCR using primers distinct from those applied in the present study [44]. The comparatively higher number of discordant isolates observed in the present study (*n* = 16), predominantly *S. epidermidis* (*n* = 11), further supports the hypothesis that false-negative PCR results attributable to primer limitations may account for a substantial proportion of these cases, underscoring the need for primer validation in CoNS species prior to molecular epidemiological studies.

Statistical analysis identified significant associations between species and antibiotic resistance, including resistance to penicillin, cefoxitin, ciprofloxacin, gentamicin, and trimethoprim–sulfamethoxazole. This is consistent with reports that the resistance to various antimicrobial agents is not necessarily a consequence of specific CoNS species but rather reflects a trend of resistance genes being found across species [45].

The association between mupirocin, clindamycin, and trimethoprim–sulfamethoxazole suggests these determinants may be collocated on a common mobile genetic element, consistent with previously described co-selection of resistance genes on plasmid or integrons in CoNS [46]. A similar correlation was observed between resistance to erythromycin and clindamycin. The classic genetic explanation for this pattern, particularly for the inducible phenotype, is the presence of *erm* genes (most commonly *erm*C in staphylococci), which encode ribosomal methylases conferring cross-resistance to macrolides, lincosamides, and streptogramin B; expression of these genes can be inducible, conferring resistance to lincosamides only in the presence of a macrolide inducer [46]. The D-test revealed that 12.1% of the CoNS isolates exhibited this inducible phenotype, confirming that a proportion of the isolates can express resistance to clindamycin exclusively in the presence of macrolides, a finding of clinical importance given the risk of therapeutic failure if clindamycin is used without prior D-test screening. However, erm genes were not investigated in the present study, which examined *mph*C and *msr*A/B as markers of macrolide resistance. These genes encode efflux and phosphotransferase mechanisms that do not typically explain lincosamide cross-resistance or the inducible phenotype. The genetic basis of the constitutive and inducible resistance patterns observed here, therefore, remains to be confirmed. This is consistent with findings by Szemraj et al. (2019), who examined MLSB resistance genes, including *erm*(A), *erm*(B), and *erm*(C), across the same predominant CoNS species as those in the present study (*S. epidermidis*, *S. hominis*, *S. haemolyticus*, *S. warneri*), and reported that these genes were detected only infrequently despite a high phenotype prevalence of the MLSB mechanism, indicating that genotype-phenotype discordance in this context is not unique to the present dataset [47]. Previous studies have reported varying prevalences of inducible resistance in CoNS, depending on the species and genetic profile of the isolates [48]. A similar correlation was observed between resistance to clindamycin and trimethoprim–sulfamethoxazole, suggesting a potential co-selection of these resistance determinants, as previously described in CoNSs [49]. The strong correlation between gentamicin, tobramycin, and kanamycin reflects the expected cross-resistance within the aminoglycoside class, given shared enzymatic resistance mechanisms [50,51]. The positive correlation between ciprofloxacin and cefoxitin suggests that fluoroquinolone resistance may share genetic mechanisms with β-lactam resistance [52]. The absence of correlation for the remaining antibiotics suggests these resistance mechanisms occur independently in this collection.

The biofilm-forming capacity of CoNS isolates in this study varied considerably, with 64.9% classified as super-producers. Reported strong or high biofilm producer rates in other CoNS studies range widely from 9.8% to 51.4%, with the lowest values reported in isolates from hospital environmental surfaces rather than clinical specimens [53,54,55,56]. These comparisons should be interpreted with caution, as biofilm classification schemes vary considerably across studies in terms of reference control (positive versus negative), quantification method, and threshold definitions, meaning the percentages reported are not necessarily on a directly equivalent scale.

The lack of significant differences in biofilm production among species, per ANOVA, suggests that biofilm formation is not an intrinsic species-specific trait but rather a consequence of genetic and environmental factors, consistent with Silva et al. (2022), who proposed that biofilm development is influenced more by external conditions than by species identity [57].

Mann–Whitney U testing showed no significant correlation between biofilm production and antibiotic resistance, in line with reports that these traits are not necessarily governed by the same mechanisms [56]. This contrasts with studies reporting that biofilm formation increases antibiotic resistance [58] but is consistent with other findings that show no direct correlation [59].

This study has some limitations that should be acknowledged. Patient-level clinical information, including the presence of central venous catheters, prosthetic devices, or other recognized risk factors for CoNS infection, was not accessible for this study, as data sharing with the partner hospital was limited to microbiological and antimicrobial susceptibility data, in accordance with patient data protection requirements. Although all isolates included had been classified as clinically significant by the hospital’s clinical microbiology laboratory, the specific criteria underlying this classification were not accessible to the authors, limiting further characterization of the clinical context of individual isolates.

These findings carry clinical epidemiological relevance. The high prevalence of multidrug-resistant CoNS underscores the need for strict infection control in hospital settings, where these bacteria act as reservoirs of resistance genes, while the substantial proportion of biofilm-producing isolates highlights the challenge of managing biofilm-associated infections refractory to standard treatment. The association between patient gender and CoNS species distribution warrants further investigation into its underlying determinants.

## 4. Materials and Methods

### 4.1. Study Design and Sampling

This study analyzed 148 clinical isolates of CoNS from patients at the Hospital Center of Trás-os-Montes and Alto Douro in Portugal, collected between November 2023 and June 2024. Specimens were collected from different clinical sources, predominantly blood and urine, with occasional isolates from wound swabs, drainage fluid, blood culture broth, pus, and bronchial aspirate. All isolates had been classified by the hospital’s clinical microbiology laboratory as clinically significant, as opposed to probable contamination, at the time of processing, and only isolates in the former category were included in this study. Repeat isolates of the same species recovered from the same patient were excluded, ensuring that each isolate represents a distinct patient-species combination.

### 4.2. Sample Identification and Characterization

The bacterial isolates were identified by conventional microbiological methods, including the use of selective media and biochemical tests. These were then confirmed by MALDI-TOF analysis at the Hospital Center of Trás-os-Montes and Alto Douro.

### 4.3. Antimicrobial Susceptibility Test

The antimicrobial susceptibility test was performed using the Kirby–Bauer disk diffusion method, following the 2024 guidelines of the European Committee for Antimicrobial Susceptibility Testing (EUCAST), except for kanamycin, which followed the 2024 standards of the Clinical and Laboratory Standards Institute (CLSI). A total of 14 antibiotics were tested: penicillin G (1U), cefoxitin (30 μg), gentamicin (10 μg), tobramycin (10 μg), tetracycline (30 μg), chloramphenicol (30 μg), erythromycin (15 μg), clindamycin (2 μg), ciprofloxacin (5 μg), linezolid (10 μg), trimethoprim–sulfamethoxazole (1.25/23.75 μg), fusidic acid (10 μg), kanamycin (30 μg) and mupirocin (200 μg). For cefoxitin, ciprofloxacin, tetracycline, trimethoprim–sulfamethoxazole, and linezolid, susceptibility results were primarily obtained from the routine Vitek 2 system results reported by the partner hospital’s clinical microbiology laboratory, interpreted using EUCAST breakpoints, and reflecting the antibiotic panel requested by the attending physician for each patient. For isolates lacking a Vitek result for one or more of these anabiotics, susceptibility was determined in the authors’ laboratory using the Kirby–Bauer disk diffusion method described above, ensuring a complete susceptibility profile for all 148 isolates. Susceptibility to penicillin G, tobramycin, kanamycin, chloramphenicol, fusidic acid, and mupirocin was determined by disk diffusion for the entire isolate collection. The reference strain *S. epidermidis* ATCC^®^ 35984 was used as a quality control strain. CoNS isolates that exhibited resistance to erythromycin but remained susceptible to clindamycin were selected for (macrolide–lincosamide–streptogramin B) MLS_B phenotype screening using the disk approximation test (D-test), as previously described by [9]. In brief, a 15 μg erythromycin disk was placed 15–20 mm apart from a 2 μg clindamycin disk in Mueller–Hinton agar plates. If the presence of a flattened “D-shaped” inhibition zone between the disk was observed, it was considered to be positive for inducible resistance.

### 4.4. Molecular Characterization

#### 4.4.1. DNA Extraction

DNA was extracted using the boiling method. Bacterial colonies were suspended in 200 μL of sterile distilled water and incubated at 100 °C for 10 min, followed by a heat shock at −20 °C for 5 min. The mixture was then centrifuged at 12,000 rpm for 10 min, after which the pellet was discarded. The obtained DNA was stored at −20 °C.

#### 4.4.2. Antimicrobial Resistance Genes

The presence of antimicrobial resistance genes was determined by PCR, based on antibiotic resistance phenotypes, as described in Table S3. The following resistance genes were analyzed: β-lactams (*mec*A, *mec*C, *bla*Z), macrolides and lincosamides (*mph*C, *msr*A/B), tetracyclines (*tet*O), aminoglycosides (*aac*(6′)-Ie-*aph*(2″)-Ia), chloramphenicol (*cat*_PC194_, *cat*_PC221_, *cat*_PC223_, *fex*A, *fex*B), mupirocin (*mup*A), trimethoprim–sulfamethoxazole (*dfr*A, *dfr*D, *dfr*G, *dfr*K), and linezolid (*cfr*). The positive controls used in all tests were from the strain collection of the Microbiology and Antibiotic Resistance Team (MicroART) research group at the University of Trás-os-Montes e Alto Douro.

### 4.5. Biofilm Formation Assay

The microtiter assay was used to assess biofilm formation, following the method described by Oniciuc et al. (2016) with modifications. Each CoNS isolate was seeded onto BHI agar plates and incubated at 37 °C for 24 h [60]. Two Staphylococcus colonies were then transferred to tubes containing 3 mL of tryptic soy broth (TSB) and incubated at 37 °C for 16 ± 1 h with continuous shaking at 150 rpm. The bacterial suspension was adjusted to an optical density corresponding to 1 × 10^6^ colony-forming units, and 200 μL of this suspension was added to each well of a 96-well flat-bottom microplate. *S. epidermidis* ATCC^®^ 35984 was included as a positive control, while TSB without bacterial inoculum was used as a negative control. The plates were incubated at 37 °C for 24 h under strictly aerobic conditions, without external stirring. Each experiment was performed in duplicate, with seven technical replicates.

#### Biofilm Biomass Quantification

Biofilm biomass was quantified using the crystal violet (CV) staining method, as described by Peeters et al. (2008) [61], with modifications. After incubation, the medium was carefully removed, and the plates were washed twice with distilled water to eliminate non-adherent bacterial cells. The plates were air-dried at room temperature for two hours. To fix the biofilms, 100 μL of methanol was added to each well and incubated for 15 min. After removal of the methanol, the plates were left to air dry for 10 min before the addition of 100 μL of 1% (*v*/*v*) CV for 10 min. Excess stain was removed by washing the plates twice with distilled water. Finally, 100 μL of 33% (*v*/*v*) acetic acid was added to solubilize the CV, and absorbance was measured at 570 nm using a microplate reader. The reference strain *S. epidermidis* ATCC^®^ 35984 was included as a positive control on every plate tested, and biofilm formation for each isolate was expressed as a percentage of the OD570 value obtained for the reference strain on the same plate. Isolates exceeding the reference OD570 value (>100%) were classified as super-producers, isolates between 50% and 100% as producers, and isolates below 50% as non-producers. These thresholds were established for the purpose of this study.

### 4.6. Statistical Analysis

Statistical analysis of categorical and numeric variables was conducted using the SPSS software package, version 29.0.2.0 (SPSS Inc., Chicago, IL, USA). Pearson’s chi-square test, ANOVA, Mann–Whitney U test, and Pearson’s correlation were applied. A *p*-value < 0.05 was considered statistically significant.

## 5. Conclusions

This study provides valuable data on the antimicrobial resistance patterns, genotypic determinants, and biofilm-forming capacity. The findings underscore the clinical significance of CoNSs in healthcare-associated infections and highlight the need for ongoing surveillance, targeted therapeutic approaches, and infection control measures to mitigate their impact. Further research is necessary to fully understand the genetic and environmental factors driving resistance and biofilm formation in these opportunistic pathogens.

## Figures and Tables

**Table 1:** Results of the chi-square test to calculate the association between species and antibiotic resistance. *p* < 0.05 indicates a statistically significant association; *p* < 0.01 indicates a highly significant association. Cramér’s V was not calculated (“/”) for antibiotics without a statistically significant association.

Antibiotic	Chi-Square	*p*-Value	Cramér’s V
Penicillin	39.754	<0.001	0.518
Cefoxitin	29.174	0.001	0.444
Ciprofloxacin	28.524	0.001	0.439
Gentamicin	36.648	<0.001	0.498
Tobramycin	30.621	<0.001	0.455
Kanamycin	29.291	0.001	0.445
Erythromycin	23.379	0.009	0.397
Clindamycin	51.4	<0.001	0.417
Tetracycline	11.605	0.312	/
Chloramphenicol	1.034	1.000	/
Fusidic Acid	11.998	0.285	/
SXT	32.793	<0.001	0.474
Mupirocin	15.249	0.123	/

## Data Availability

The original contributions presented in the study are included in the article/Supplementary Materials; further inquiries can be directed to the corresponding authors.

## Supplementary Materials

The following supporting information can be downloaded at: https://www.mdpi.com/article/10.3390/pathogens15070745/s1. Table S1: Antimicrobial resistance phenotype and genotype of CoNSs isolated; Table S2: Correlation between antibiotic resistance profiles in coagulase-negative Staphylococcus (CoNS) isolates; Table S3: Primer pairs used for molecular typing and detection of antimicrobial resistance genes, adapted from [43,62,63,64,65,66,67,68,69,70].
