# Supplementary material for: Multidrug Resistance and mupA-Mediated Mupirocin Resistance in Clinical Coagulase-Negative Staphylococci
Source: Pathogens. 2026 Jul 15;15(7):745. doi: 10.3390/pathogens15070745 (PMC13416171; doi:10.3390/pathogens15070745)
Supplement: Supplementary file 1 [file pathogens-15-00745-s001.zip › pathogens-4384815-supplementary.pdf]

Table S1. Antimicrobial resistance phenotype and genotype of CoNS isolated. N.F. Not found; N.D. Not determined.

Isolate	CoNS Species	Antimicrobial Resistance	
		Phenotype	Genotype
CF0056	*S. simulans*	FC	N.D.
CF0057	*S. saprophyticus*	P1, TET, FC	N.F.
CF0058	*S. schleiferi*	N.F	N.D.
CF0059	*S. warneri*	CD, FC	N.F.
CF0060	*S. caprae*	P1, FOX, CIP, CN, TOB, K, ERY, CD	*mec*A, *bla*Z, *aac*(6′)-Ie-*aph*(2′′)-Ia
CF0061	*S. hominis*	P1, FOX, CN, TOB, K, ERY, SXT	*mec*A, *bla*Z, *msr*A/B, *aac*(6′)-Ie-*aph*(2′′)-Ia
CF0062	*S. epidermidis*	P1, FOX, ERY	*mec*A, *msr*A/B
CF0063	*S. epidermidis*	P1, FOX, TOB, ERY, CD, TET	*mec*A
CF0064	*S. haemolyticus*	P1, FOX, CIP, CN, TOB, K, ERY, CD, TET, FC, SXT, MUP	*mec*A, *msr*A/B, *aac*(6′)-Ie-*aph*(2′′)-Ia, *mup*A, *dfr*G
CF0065	*S. epidermidis*	N.F	N.D.
CF0066	*S. epidermidis*	P1, ERY, TET, FC	*msr*A/B
CF0067	*S. haemolyticus*	P1, FOX, CIP, CN, TOB, K, ERY, CD, TET, SXT, MUP	*mec*A, *msr*A/B, *aac*(6′)-Ie-*aph*(2′′)-Ia, *mup*A, *dfr*G
CF0068	*S. epidermidis*	P1, FOX, CIP, CN, TOB, K, ERY, CD, FC, SXT	*aac*(6′)-Ie-*aph*(2′′)-Ia, *dfr*A
CF0069	*S. epidermidis*	P1, CN, TET, SXT	*dfr*A
CF0070	*S. epidermidis*	P1, FOX, CIP, ERY, CD, SXT	*mec*A, *dfr*A
CF0071	*S. hominis*	P1, FOX, CIP, CN, TOB, K, ERY, CD, SXT, MUP	*mec*A, *aac*(6′)-Ie-*aph*(2′′)-Ia, *mup*A
CF0072	*S. epidermidis*	P1, FOX, CIP, CN, TOB, K, ERY, CD, TET, SXT, MUP	*mec*A, *aac*(6′)-Ie-*aph*(2′′)-Ia, *mup*A, *dfr*A
CF0073	*S. epidermidis*	P1, FOX, CIP, CN, TOB, K, ERY, CD, SXT, MUP	*dfr*A
CF0074	*S. epidermidis*	ERY, FC	N.F.
CF0075	*S. hominis*	ERY,CD	N.F.
CF0076	*S. lugdunensis*	P1, LNZ	N.F.
CF0077	*S. epidermidis*	P1, FOX, CIP, ERY, CD, TET, SXT	*mec*A, *dfr*A
CF0078	*S. epidermidis*	P1, FOX, ERY, TET	N.F.
CF0079	*S. epidermidis*	P1, FOX, CIP, CN, TOB, K, ERY, CD, TET, SXT	*mec*A, *aac*(6′)-Ie-*aph*(2′′)-Ia, *dfr*A
CF0080	*S. epidermidis*	P1, FOX, LNZ, FC, MUP	N.F.
CF0081	*S. epidermidis*	P1, FOX, CIP, ERY, CD, SXT	*mec*A, *dfr*A
CF0082	*S. epidermidis*	P1, FOX, CN, TOB, K, ERY, FC	*mec*A, *aac*(6′)-Ie-*aph*(2′′)-Ia
CF0083	*S. hominis*	P1, FOX, ERY, FC	*mec*A
CF0084	*S. epidermidis*	P1, FOX, CIP, CN, TOB, K, ERY, CD, SXT, MUP	*mec*A, *aac*(6′)-Ie-*aph*(2′′)-Ia, *dfr*A
CF0085	*S. capitis*	N.F	N.D.
CF0086	*S. hominis*	P1, FOX, CIP, ERY, SXT	m*ec*A, *msr*A/B
CF0087	*S. hominis*	P1, K, FC	N.F.
CF0088	*S. epidermidis*	P1, FOX, CIP, ERY, CD, SXT,	*mec*A, *dfr*A

CF0089	*S. epidermidis*	P1, FOX, CIP, CN, TOB, K, ERY, CD, FC, SXT	*mec*A, *aac*(6′)-Ie-*aph*(2′′)-Ia, *dfr*A
CF0090	*S. capitis*	P1, FOX, ERY, FC	*mec*A
CF0091	*S. hominis*	P1, ERY, TET	*mph*C, *msr*A/B
CF0092	*S. saprophyticus*	P1, FOX, ERY, TET, FC	*mec*A, *msr*A/B
CF0093	*S. hominis*	P1, FOX, CIP, CN, TOB, K, ERY, CD, FC, SXT	*mec*A, *aac*(6′)-Ie-*aph*(2′′)-Ia
CF0094	*S. hominis*	P1, FOX, CIP, CN, TOB, K, ERY, CD, FC	m*ec*A, *aac*(6′)-Ie-*aph*(2′′)-Ia
CF0095	*S. epidermidis*	P1, FOX, CN, TOB, K, ERY, CD, FC, SXT	*mec*A, *bla*Z, *aac*(6′)-Ie-*aph*(2′′)-Ia, *dfr*A
CF0096	*S. capitis*	P1, FC	*bla*Z
CF0097	*S. epidermidis*	P1, FOX, TET, FC	*mec*A, *bla*Z
CF0098	*S. hominis*	P1, FOX, TOB, ERY, TET, FC, SXT	*mec*A, *bla*Z, *msr*A/B
CF0099	*S. haemolyticus*	P1, FOX, CIP, CN, TOB, K, ERY, CD, FC, SXT	*mec*A, *bla*Z, *aac*(6′)-Ie-*aph*(2′′)-Ia, *dfr*G
CF0100	*S. hominis*	P1, FOX, CIP, TOB, K, ERY, FC, SXT, MUP	*mec*A, *aac*(6′)-Ie-*aph*(2′′)-Ia
CF0101	*S. epidermidis*	P1, FOX, CIP, CN, TOB, K, ERY, CD, FC, SXT	*mec*A, *bla*Z, *aac*(6′)-Ie-*aph*(2′′)-Ia, *dfr*A
CF0102	*S. epidermidis*	P1, FOX, ERY, FC	N.F.
CF0103	*S. hominis*	P1, FOX, CN, FC, SXT	*mec*A
CF0104	*S. epidermidis*	P1, FC	N.F.
CF0105	*S. haemolyticus*	P1, FOX, IP, CN, TOB, K, ERY, CD, FC, SXT, MUP	*mec*A, *msr*A/B, *aac*(6′)-Ie-*aph*(2′′)-Ia, *dfr*G
CF0106	*S. capitis*	P1, CIP, FC	*mec*A
CF0107	*S. capitis*	P1, FOX, ERY, FC	*mec*A, *msr*A/B
CF0108	*S. epidermidis*	P1, FOX, ERY, TET, FC	*mec*A
CF0109	*S. haemolyticus*	P1, FOX, CIP, CN, TOB, K, ERY, CD, SXT, MUP	*mec*A, *aac*(6′)-Ie-*aph*(2′′)-Ia, *mup*A, *dfr*G
CF0110	*S. epidermidis*	P1, FOX, FC	N.F.
CF0111	*S. epidermidis*	P1, FOX, CIP, TET, FC, SXT	*mec*A, *bla*Z, *dfr*A
CF0112	*S. epidermidis*	P1, FOX, CIP, CN, TOB, K, CD, FC, SXT	*mec*A, *bla*Z, *aac*(6′)-Ie-a*ph*(2′′)-Ia, *dfr*A
CF0113	*S. epidermidis*	P1, FOX, CIP, CN, TOB, K, ERY, CD, TET, SXT, MUP	*mec*A, *aac*(6′)-Ie-*aph*(2′′)-Ia, *dfr*A
CF0114	*S. hominis*	P1, FOX, CN, K, TET, FC	*bla*Z, *aac*(6′)-Ie-*aph*(2′′)-Ia
CF0115	*S. epidermidis*	P1	N.F.
CF0116	*S. epidermidis*	P1, FOX, CIP, K, ERY, FC	*mec*A, *bla*Z, *msr*A/B
CF0117	*S. epidermidis*	P1, FOX, CIP, CN, TOB, K, SXT	*mec*A, *aac*(6′)-Ie-*aph*(2′′)-Ia, *dfr*A
CF0118	*S. hominis*	P1, FOX, ERY, FC	*mec*A
CF0119	*S. hominis*	P1, FOX, ERY, FC, SXT	*mec*A, *bla*Z, *dfr*A
CF0120	*S. hominis*	P1, FOX	*mec*A
CF0121	*S. epidermidis*	P1, FOX, CIP, CN, TOB, K, ERY, CD, FC, MUP	*mec*A, *msr*A/B, *aac*(6′)-Ie-*aph*(2′′)-Ia, *dfr*G
CF0122	*S. hominis*	TET	N.F.
CF0123	*S. epidermidis*	P1, FOX, CIP, CN, TOB, K, ERY, CD, TET, FC, SXT	*mec*A, *bla*Z, *aac*(6′)-Ie-*aph*(2′′)-Ia, *dfr*A

CF0124	*S. hominis*	P1, TOB, ERY	*bla*Z
CF0125	*S. epidermidis*	P1, FOX, CIP, CN, TOB, K, ERY, CD, FC, SXT	*mec*A, *bla*Z, *aac*(6′)-Ie-*aph*(2′′)-Ia, *dfr*A
CF0126	*S. epidermidis*	P1, FOX, CIP, CN, TOB, K, ERY, CD, FC, SXT	*mec*A, *aac*(6′)-Ie-*aph*(2′′)-Ia, *dfr*A
CF0127	*S. epidermidis*	P1, FOX, TOB, ERY, TET	*mec*A, *mph*C, *msr*A/B
CF0128	*S. hominis*	P1, FOX, CIP, ERY, FC, SXT	*mec*A, *mph*C, *msr*A/B
CF0129	*S. epidermidis*	P1, ERY, FC	N.F.
CF0130	*S. epidermidis*	P1, FOX, CN, TOB, K, ERY, CD, TET, FC, SXT	*mec*A, *aac*(6′)-Ie-*aph*(2′′)-Ia, *dfr*A
CF0131	*S. epidermidis*	P1, FOX, CIP, ERY, CD, FC, SXT	*mec*A, *bla*Z, *dfr*A
CF0132	*S. epidermidis*	P1, FOX, CIP, CN, TOB, K, ERY, CD, FC, SXT	*mec*A, *aac*(6′)-Ie-*aph*(2′′)-Ia, *dfr*A
CF0133	*S. hominis*	ERY, TET, FC	*mph*C, *msr*A/B
CF0134	*S. epidermidis*	P1, FOX, CIP, CN, TOB, K, ERY, CD, TET, SXT, MUP	*mec*A, *aac*(6′)-Ie-*aph*(2′′)-Ia, *dfr*A
CF0135	*S. haemolyticus*	P1, FOX, CIP, CN, TOB, K, ERY, CD, TET, SXT, MUP	*mec*A, *bla*Z, *mph*C, *msr*A/B, *aac*(6′)-Ie-*aph*(2′′)-Ia, *mup*A, *dfr*G
CF0136	*S. capitis*	N.F	N.D.
CF0137	*S. hominis*	P1, TET, FC	*bla*Z
CF0138	*S. epidermidis*	P1, FOX, ERY, CD, FC	N.F.
CF0139	*S. epidermidis*	P1, FOX, ERY, FC	*mec*A, *msr*A/B
CF0140	*S. epidermidis*	P1, TOB, ERY, CD, FC	*mec*A
CF0141	*S. simulans*	CIP	N.D.
CF0142	*S. hominis*	P1, FOX, CIP, TOB, ERY, TET, FC, SXT	*mec*A, *bla*Z
CF0143	*S. epidermidis*	P1, FC	N.F.
CF0144	*S. hominis*	P1, FOX, CIP, CN, TOB, K, ERY, CD, SXT, MUP	*mec*A, *aac*(6′)-Ie-*aph*(2′′)-Ia, *mup*A
CF0145	*S. hominis*	P1, K, ERY, FC	*mec*A, *bla*Z, *mph*C, *msr*A/B
CF0146	*S. hominis*	P1, FOX, CN, TOB, K, ERY, TET, FC	*mec*A, *msr*A/B, *aac*(6′)-Ie-*aph*(2′′)-Ia
CF0147	*S. hominis*	P1, FOX, CN, TOB, K, ERY, CD, TET, FC	*mec*A, *aac*(6′)-Ie-*aph*(2′′)-Ia
CF0148	*S. epidermidis*	P1, FOX, CIP, CN ,TOB, K, ERY, CD, FC, SXT	*mec*A, *aac*(6′)-Ie-*aph*(2′′)-Ia, *dfr*A
CF0149	*S. epidermidis*	CN, TOB, FC	N.F.
CF0150	*S. haemolyticus*	P1, FOX, CIP, CN, TOB, K, ERY, CD, SXT	*mec*A, *msr*A/B, *aac*(6′)-Ie-*aph*(2′′)-Ia, *dfr*G
CF0151	*S. hominis*	P1, TET, FC	*bla*Z
CF0152	*S. epidermidis*	P1, FOX, CIP, CN, TOB, K, ERY, CD, TET, FC, SXT, MUP	*aac*(6′)-Ie-*aph*(2′′)-Ia
CF0153	*S. epidermidis*	FOX, TOB, K ,ERY, CD, FC	*mec*A, *msr*A/B
CF0154	*S. epidermidis*	P1, FOX, CN, TOB, K, ERY, CD, FC	N.F.
CF0155	*S. hominis*	P1, ERY, FC	N.F.
CF0156	*S. haemolyticus*	P1, CIP, CN, TOB, K, ERY, FC, SXT	*mec*A, *msr*A/B, *aac*(6′)-Ie-*aph*(2′′)-Ia, *dfr*G

CF0157	*S. epidermidis*	P1, FOX, CN, TOB, K, ERY, CD, FC, SXT	*mec*A, *aac*(6′)-Ie-*aph(*2′′)-Ia, *dfr*A
CF0158	*S. hominis*	P1, FOX, CIP, CN, TOB, K, ERY, CD, SXT, MUP	*mec*A, *aac*(6′)-Ie-*aph*(2′′)-Ia
CF0159	*S. epidermidis*	P1, FOX, CIP, CN, TOB, K, ERY, CD, SXT	*mec*A, *aac*(6′)-Ie-*aph*(2′′)-Ia, *dfr*A
CF0160	*S. epidermidis*	P1, FOX, CIP, ERY, CD, FC,SXT	*mec*A, *dfr*A
CF0161	*S. epidermidis*	P1, CIP, CN, TOB, K, FC, SXT	*aac*(6′)-Ie-*aph(*2′′)-Ia, *dfr*A
CF0162	*S. epidermidis*	P1, FOX, CIP, CN, TOB, K, ERY, CD, TET, FC, SXT	*dfr*A
CF0163	*S. hominis*	ERY	N.F.
CF0164	*S. hominis*	P1, FOX, CIP, ERY, FC	*mec*A
CF0165	*S. epidermidis*	P1, CIP, CN, TOB, K, ERY, CD, FC, SXT	*mec*A, *aac*(6′)-Ie-*aph*(2′′)-Ia, *dfr*A
CF0166	*S. saprophyticus*	TET	N.F.
CF0167	*S. epidermidis*	P1, CIP, CN, TOB, K, ERY, CD, FC	*mec*A, *bla*Z, *aac*(6′)-Ie-*aph*(2′′)-Ia
CF0168	*S. petrasii*	P1, ERY	N.F.
CF0169	*S. epidermidis*	P1, FOX, CIP, CN, TOB, K, ERY, CD, TET, SXT, MUP	*mec*A, *aac*(6′)-Ie-*aph*(2′′)-Ia, *dfr*A
CF0170	*S. epidermidis*	P1, FOX, CIP, ERY, CD, TET, SXT	*mec*A, *dfr*A
CF0171	*S. hominis*	P1, FC	*bla*Z
CF0172	*S. capitis*	P1	N.F.
CF0173	*S. hominis*	FOX, ERY, FC	N.F.
CF0174	*S. epidermidis*	P1, FOX, CIP, CN, TOB, K, ERY, CD, C, FC	*mec*A, *aac*(6′)-Ie-*aph*(2′′)-Ia
CF0175	*S. saprophyticus*	FC	N.D.
CF0176	*S. hominis*	P1, TOB, TET, FC	N.F.
CF0177	*S. epidermidis*	P1, FOX, CIP, ERY, CD, TET, SXT	*mec*A, *dfr*A
CF0178	*S. hominis*	N.F	N.D.
CF0179	*S. epidermidis*	P1, FOX, ERY, CD, FC	*msr*A/B
CF0180	*S. epidermidis*	N.F	N.D.
CF0181	*S. epidermidis*	P1, FOX, CIP, ERY, CD, TET, SXT	*mec*A, *dfr*A
CF0182	*S. epidermidis*	P1, CN, ERY, TET, FC	*msr*A/B
CF0183	*S. saprophyticus*	ERY, CD	N.F.
CF0184	*S. capitis*	P1, FOX, CIP, TOB, K, ERY, CD, FC, MUP	*mec*A, *msr*A/B, *aac*(6′)-Ie-*aph*(2′′)-Ia
CF0185	*S. capitis*	P1, FOX, ERY, CD, FC, SXT	N.F.
CF0186	*S. epidermidis*	P1, FOX,, CIP, CN, TOB, K, ERY, CD, TET, FC, SXT	*mec*A, *aac*(6′)-Ie-*aph*(2′′)-Ia, *dfr*A
CF0187	*S. hominis*	P1, FOX, CIP, TOB, K, ERY, FC, SXT, MUP	*mec*A, *bla*Z, *aac*(6′)-Ie-*aph*(2′′)-Ia, *mup*A
CF0188	*S. epidermidis*	P1, FOX, CIP, CN, TOB, K, SXT	*mec*A, *aac*(6′)-Ie-*aph*(2′′)-Ia, *dfr*A
CF0189	*S. hominis*	P1, FOX, CIP, CN, TOB, K, ERY, SXT, MUP	*mec*A, *aac*(6′)-Ie-*aph*(2′′)-Ia, *mup*A
CF0190	*S. caprae*	N.F	N.D.
CF0191	*S. hominis*	P1	*mec*A

CF0192	*S. hominis*	P1, FOX, CIP, TOB, K, ERY, TET, FC, SXT, MUP	*mec*A
CF0193	*S. haemolyticus*	P1, FOX, CIP, CN, TOB, K, ERY, CD, TET, SXT, MUP	*mec*A, *bla*Z, *msr*A/B, *aac*(6′)-Ie-*aph*(2′′)-Ia, *mup*A, *dfr*G
CF0194	*S. hominis*	P1, FOX, CIP, ERY, FC	*mec*A
CF0195	*S. warneri*	FOX, ERY, FC	N.F.
CF0196	*S. epidermidis*	P1, FOX, CIP, TOB, K, ERY, TET, FC, SXT, MUP	*mec*A
CF0197	*S. epidermidis*	P1, FOX, CIP, CN, TOB, K, ERY, CD, SXT, MUP	*mec*A, *aac*(6′)-Ie-*aph*(2′′)-Ia, *dfr*A
CF0198	*S. capitis*	P1, CD	N.F.
CF0199	*S. saprophyticus*	ERY	*msr*A/B
CF0200	*S. epidermidis*	FOX, CN, ERY, CD, FC, SXT	*dfr*A
CF0201	*S. epidermidis*	P1, FOX, CIP, CN, TOB, K, ERY, CD, TET, FC, SXT, MUP	*mec*A, *aac*(6′)-Ie-*aph*(2′′)-Ia, *dfr*A
CF0202	*S. warneri*	P1, ERY, FC	*bla*Z, *msr*A/B
CF0203	*S. epidermidis*	P1, FOX, ERY, CD	*mec*A, *msr*A/B

Table S2. Correlation between antibiotic resistance profiles in coagulase-negative Staphylococcus (CoNS) isolates. The analysis was carried out using Pearson's correlation coefficient. Values of $p < 0.05$ indicate statistically significant correlations. ** Correlation is significant at the 0.01 level (2 ends). * Correlation is significant at the 0.05 level (2 ends).

		Biofilm	Erythromycin	Clindamycin	Tetracycline	Linezolid	Chloramphenicol	FusidicAcid	SXT	Mupirocin	Penicillin	Cefoxitin	Ciprofloxacin	Gentamicin	Tobramycin	Kanamycin
**Biofilm**	Pearson's correlation (r)	1	-0,070	-0,066	0,094	-0,027	-0,004	-0,013	-0,120	-0,088	-0,049	-0,069	0,036	-0,056	-0,012	0,036
	p		0,398	0,423	0,256	0,743	0,957	0,877	0,145	0,286	0,558	0,406	0,660	0,498	0,888	0,668
**Erythromycin**	Pearson's correlation (r)	-0,070	1	,472**	0,081	-,189*	0,051	0,091	,351**	0,133	,301**	,547**	,359**	,257**	,381**	,350**
	p	0,398		0,000	0,328	0,021	0,538	0,273	0,000	0,107	0,000	0,000	0,000	0,002	0,000	0,000
**Clindamycin**	Pearson's correlation (r)	-0,066	,472**	1	0,019	-0,106	0,091	-0,104	,536**	,266**	,253**	,449**	,551**	,563**	,509**	,507**
	p	0,423	0,000		0,823	0,198	0,273	0,208	0,000	0,001	0,002	0,000	0,000	0,000	0,000	0,000
**Tetracycline**	Pearson's correlation (r)	0,094	0,081	0,019	1	-0,077	-0,055	-0,041	0,137	,165*	0,131	0,131	0,039	0,062	0,137	0,055
	p	0,256	0,328	0,823		0,350	0,510	0,620	0,098	0,045	0,111	0,114	0,638	0,455	0,098	0,508
**Linezolid**	Pearson's correlation (r)	-0,027	-,189*	-0,106	-0,077	1	-0,010	-0,026	-0,106	0,084	0,051	-0,040	-0,108	-0,095	-0,106	-0,101
	p	0,743	0,021	0,198	0,350		0,907	0,755	0,198	0,313	0,534	0,628	0,192	0,249	0,198	0,223
**Chloramphenicol**	Pearson's correlation (r)	-0,004	0,051	0,091	-0,055	-0,010	1	0,066	-0,075	-0,042	0,036	0,059	0,089	0,101	0,091	0,096
	p	0,957	0,538	0,273	0,510	0,907		0,424	0,365	0,608	0,662	0,477	0,280	0,221	0,273	0,247
**FusidicAcid**	Pearson's correlation (r)	-0,013	0,091	-0,104	-0,041	-0,026	0,066	1	-0,076	-,165*	,173*	0,100	-0,065	0,032	0,063	0,103
	p	0,877	0,273	0,208	0,620	0,755	0,424		0,357	0,045	0,036	0,228	0,430	0,702	0,449	0,212
**SXT**	Pearson's correlation (r)	-0,120	,351**	,536**	0,137	-0,106	-0,075	-0,076	1	,299**	,363**	,535**	,714**	,563**	,536**	,535**
	p	0,145	0,000	0,000	0,098	0,198	0,365	0,357		0,000	0,000	0,000	0,000	0,000	0,000	0,000
**Mupirocin**	Pearson's correlation (r)	-0,088	0,133	,266**	,165*	0,084	-0,042	-,165*	,299**	1	0,091	,227**	,358**	,259**	,366**	,396**
	p	0,286	0,107	0,001	0,045	0,313	0,608	0,045	0,000		0,270	0,005	0,000	0,001	0,000	0,000
**Penicillin**	Pearson's correlation (r)	-0,049	,301**	,253**	0,131	0,051	0,036	,173*	,363**	0,091	1	,461**	,369**	,283**	,326**	,342**
	p	0,558	0,000	0,002	0,111	0,534	0,662	0,036	0,000	0,270		0,000	0,000	0,000	0,000	0,000
**Cefoxitin**	Pearson's correlation (r)	-0,069	,547**	,449**	0,131	-0,040	0,059	0,100	,535**	,227**	,461**	1	,487**	,377**	,420**	,442**
	p	0,406	0,000	0,000	0,114	0,628	0,477	0,228	0,000	0,005	0,000		0,000	0,000	0,000	0,000
**Ciprofloxacin**	Pearson's correlation (r)	0,036	,359**	,551**	0,039	-0,108	0,089	-0,065	,714**	,358**	,369**	,487**	1	,495**	,551**	,605**
	p	0,660	0,000	0,000	0,638	0,192	0,280	0,430	0,000	0,000	0,000	0,000		0,000	0,000	0,000
**Gentamicin**	Pearson's correlation (r)	-0,056	,257**	,563**	0,062	-0,095	0,101	0,032	,563**	,259**	,283**	,377**	,495**	1	,757**	,806**
	p	0,498	0,002	0,000	0,455	0,249	0,221	0,702	0,000	0,001	0,000	0,000	0,000		0,000	0,000
**Tobramycin**	Pearson's correlation (r)	-0,012	,381**	,509**	0,137	-0,106	0,091	0,063	,536**	,366**	,326**	,420**	,551**	,757**	1	,837**
	p	0,888	0,000	0,000	0,098	0,198	0,273	0,449	0,000	0,000	0,000	0,000	0,000	0,000		0,000
**Kanamycin**	Pearson's correlation (r)	0,036	,350**	,507**	0,055	-0,101	0,096	0,103	,535**	,396**	,342**	,442**	,605**	,806**	,837**	1
	p	0,668	0,000	0,000	0,508	0,223	0,247	0,212	0,000	0,000	0,000	0,000	0,000	0,000	0,000	

Table S3. Primer pairs used for molecular typing and detection of antimicrobial resistance genes.

Gene (amplicon size)	Sequence (5´- 3´)	Conditions	Reference
***mec*A (527 bp)**	F: GGGATCATAGCGTCATTATTC<br>R: AACGATTGTGACACGATAGCC	94ºC 5 min 1 cycle<br>94ºC 30 s<br>55ºC 30 s 30 cycles<br>72ºC 1 min<br>72ºC 10 min 1 cycle	[62]
***bla*Z (722 bp)**	F: CAGTTCACATGCCAAAGAG<br>R: TACACTCTTGGCGGTTTC	94ºC 3 min 1 cycle<br>94ºC 1 min<br>50ºC 1 min 30 cycles<br>72ºC 2 min<br>72ºC 5 min 1 cycle	[63]
***mec*C (304 bp)**	F: GGGTTCAGCCAGATTCATTTGT<br>R: GTACTGTTGCTTCGTTCAATGG	95ºC 2 min 1 cycle<br>94ºC 30 s<br>50ºC 30 s 30 cycles<br>72ºC 30 s<br>72ºC 4 min 1 cycle	[62]
***msr*(A7B) (399 bp)**	F: GCAAATGGTGTAGGTAAGACAACT<br>R: ATCATGTGATGTAAACAAAAT	95ºC 3 min 1 cycle<br>93ºC 30 s<br>55ºC 2 min 35 cycles<br>72ºC 1 min 30s<br>72ºC 5 min 1 cycle	[64]
***mph*(C) (900 bp)**	F: ATGACTCGACATAATGAAAT<br>R: CTACTCTTTCATACCTAACTC	94ºC 3 min 1 cycle<br>94ºC 1 min<br>45ºC 1 min 30 cycles<br>72ºC 1 min<br>72ºC 5 min 1 cycle	[63]
***tet*O (615 bp)**	F: GATGGCATACAGGCACAGAC<br>F: GATGGCATACAGGCACAGAC	94ºC 1 min 1 cycle<br>94ºC 1 min<br>50ºC 1 min 30 cyles<br>72 ºC 1 min<br>72 ºC 10 min 1 cycle	[65]
***aac*(6´)-Ie-*aph*(2")-Ia (220 bp)**	F: CCAAGAGCAATAAGGGCATA<br>R: CACTATCATAACCACTACCG	94ºC 5 min 1 cycle<br>94ºC 30 s<br>60ºC 45 s 30 cycles<br>72ºC 2 min<br>72ºC 7 min 1 cycle	[66]
***fex*A (1272 bp)**	F: GTACTTGTAGGTGCAATTACGGCTGA	94ºC 1 min 1 cycle<br>94ºC 1 min<br>48ºC 2 min 34 cycles	[67]

	R: CGCATCTGAGTAGGACATAGCGTC	72ºC 3 min<br>72ºC 7 min 1 cycle	
***fex*B (816 bp)**	F: TTCCCACTATTGGTGAAAGGAT<br>R: GCAATTCCCTTTTATGGACGTT	94ºC 7 min 1 cycle<br>94ºC 1 mim<br>55ºC 1 min 30 cycles<br>72ºC 1 min<br>72ºC 10 min 1 cycle	[68]
***cat*$_{pC194}$ (570 bp)**	F: CGACTTTTAGTATAACCACAGA<br>R: GCCAGTCATTAGGCCTAT	94ºC 3 min 1 cycle<br>94ºC 1 min<br>50ºC 1 min 30 cycles<br>72ºC 1 min<br>72ºC 5 min 1 cycle	[63]
***cat*$_{pC221}$ (434 bp)**	F: ATTTATGCAATTATGGAAGTTG<br>R: TGAAGCATGGTAACCATCAC		
***cat*$_{pC223}$ (238 bp)**	F: GAATCAAATGCTAGTTTTAACTC<br>R: ACATGGTAACCATCACATAC		
***dfr*A (374 bp)**	F: CCTTGGCACTTACCAAATG<br>R: CTGAAGATTCGACTTCCC	94ºC 3 min 1 cycle<br>94ºC 1 min<br>50ºC 1 min 30 cycles<br>72ºC 1 min<br>72ºC 5 min 1 cycle	[63]
***dfr*D (582 bp)**	F: TTCTTTAATTGTTGCGATGG<br>R: TTAACGAATTCTCTCATATATATG		
***dfr*G (323 bp)**	F: TCGGAAGAGCCTTACCTGACAGAA<br>R: CCCTTTTTGGGCAAATACCTCATTCCA	94ºC 3 min 1 cycle<br>94ºC 1 min<br>58ºC 1 min 30 cycles<br>72ºC 1 min<br>72ºC 5 min 1 cycle	[69]
***dfr*K (423 bp)**	F: GAGAATCCCAGAGGATTGGG<br>R: CAAGAAGCTTTTCGCTCATAAA	94ºC 3 min 1 cycle<br>94ºC 1 min<br>56ºC 1 min 30 cycles<br>72ºC 1 min<br>72ºC 5 min 1 cycle	[69]
***mup*A (1650 bp)**	F: CCCATGGCTTACCAGTTGA<br>R: CCATGGAGCACTATCCGAA	94ºC 5 min 1 cycle<br>94ºC 3 min<br>60ºC 45 s 32 cycles<br>72ºC 1 min<br>72ºC 2 min 1 cycle	[43]
***cfr* (746 bp)**	F: TGAAGTATAAAGCAGGTTGGGAGTCA<br>R: ACCATATAATTGACCACAAGCAGC	94 ºC 3 min 1 cycle<br>94ºC 1 min<br>56 ºC 1 min 30 cycles<br>72 ºC 1 min<br>72 ºC 5 min 1 cycle	[70]
